# Gender Differences in Psychosocial Pathways to Depression and Anxiety: Cross-Sectional and Bayesian Causal Network Study

**DOI:** 10.2196/76913

**Published:** 2025-10-03

**Authors:** Han Zhang, Ye Xia, Peicai Fu, Cun Li, Ke Shi, Yuan Yang

**Affiliations:** 1 Department of Neurology and Psychiatry, Tongji Hospital, Tongji Medical College, Huazhong University of Science and Technology, Jiefang Avenue, 1095, Wuhan, 430030, China, +86 13995561816; 2 Department of Neurology, Henan Provincial People's Hospital, People's Hospital of Zhengzhou University, Zhengzhou, Henan, 450003, China

**Keywords:** depression, anxiety, gender differences, network analysis, Bayesian network

## Abstract

**Background:**

Depression and anxiety are widespread disorders with documented gender differences in symptom progression and associated psychosocial factors. However, the complex interrelationships between childhood trauma, self-esteem, social support, emotion regulation, and their gender-specific impacts on the development of depression and anxiety remain unclear.

**Objective:**

The objective of this study was to investigate the network structures of depression, anxiety, and psychosocial factors and to examine the pathways contributing to the development of depression and anxiety, with a focus on gender-specific differences.

**Methods:**

This study included 6105 participants from across China, collecting their sociodemographic characteristics and psychological scale data. Cross-sectional network analysis was used to explore the complex relationships between depression, anxiety, insomnia, somatic symptoms, childhood trauma, self-esteem, social support, and emotional regulation. Subsequently, Bayesian network analysis was used to infer potential causal pathways. Gender differences in the network structures were specifically examined.

**Results:**

Network analysis revealed strong associations among depression, anxiety, insomnia, and somatic symptoms. Network strength centrality exhibited the highest stability across overall networks (CS-C=0.75), with high predictability for depression (*R*²=72.4%) and anxiety (*R*²=64%), supporting the robustness of the model. The network structure invariance test between male and female participants was significant (*P*=.001). Furthermore, the Bayesian network analysis showed gender-specific symptom progression, where anxiety preceded depression in male participants, while depression preceded anxiety in female participants (with edges retained in nearly 100% of bootstrap samples). Self-esteem, social support, and insomnia were central nodes in female participants, whereas emotion regulation was more influential in male participants. Additionally, childhood trauma influenced depression or anxiety indirectly through self-esteem and social support in both male and female participants.

**Conclusions:**

This study presents a novel application of network analyses to delineate distinct gender-specific pathways in the development of depression and anxiety. The findings underscore insomnia, self-esteem, and social support as intervention targets for women and emotion regulation for men. Findings support gender-sensitive mental health strategies and emphasize the need for longitudinal validation.

## Introduction

Mental health disorders, including depression, anxiety, insomnia, and somatization, are major public health concerns worldwide, significantly affecting individuals’ well-being and quality of life [1-3]. These psychological symptoms do not exist in isolation but are influenced by complex interconnections among various psychosocial factors [4,5]. Understanding these interconnections is crucial for developing effective prevention and intervention strategies. Among the various risk factors, childhood trauma (CT), emotion regulation (ER), self-esteem (SES), and social support (SS) are particularly significant predictors of adult mental health problems [6-11]. These factors span different developmental periods and functional domains, ranging from early life experiences (CT) to current coping resources (ER, SS) and self-concept (SES). Defined as adverse and negative experiences during early development, CT has been consistently linked to increased vulnerability to depression and anxiety in adulthood [10,12]. Empirical studies suggested that such traumatic experiences may disrupt the functioning of the hypothalamic-pituitary-adrenal (HPA) axis, creating lasting neurobiological changes that increase susceptibility to mental health issues in later life [13,14]. Two critical mediating factors in the relationship between CT and adult psychopathology are ER and SS. ER, encompassing strategies such as cognitive reappraisal (CR) and expressive suppression (ES), can be significantly impaired by early CT [15]. Conversely, SS, which includes emotional, informational, and instrumental assistance from others, serves as a protective buffer against psychological distress [9,16,17]. However, previous studies have primarily examined the influence of 1 or 2 psychosocial factors in isolation, often using traditional statistical methods that assume linear and independent relationships, thereby limiting insights into their combined and interdependent effects on depression and anxiety.

Gender differences play a crucial role in the manifestation, progression, and underlying mechanisms of mental health disorders [18-21]. Research suggested that men and women exhibited distinct emotional regulation strategies, SS utilization patterns, and responses to CT [22-24]. Women are more likely to rely on SS as a coping mechanism, while men tend to exhibit greater difficulty in expressing emotions and seeking help [25-27]. Furthermore, the impact of CT on psychological well-being may differ across genders, potentially shaping distinct pathways to mental health disorders [22,24,28]. Although previous studies have confirmed the existence of gender differences in mental health disorders, as well as the impacts of factors such as CT and SS, the exploration of the gender-specific interactive networks and potential causal pathways formed by these factors is still insufficient. A deeper understanding of these gender-specific mental health networks could provide valuable insights for personalized prevention and intervention strategies.

Network analysis has emerged as a powerful methodological approach for studying the complex interconnections among psychological factors [29]. Unlike traditional linear statistical methods that often oversimplify relationships by assuming independence or linearity, network analysis conceptualizes mental health disorders as dynamic systems of interconnected psychological variables (nodes), where direct and indirect relationships (edges) collectively shape the overall structure. This approach allows researchers to identify central nodes (ie, key factors driving the network structure) and critical pathways that may serve as potential intervention targets. Recent advances, such as Bayesian causal networks and Extended Bayesian Information Criterion Graphical LASSO (EBICglasso)-based networks, have further enhanced the ability to infer potential causal relationships and model high-dimensional psychological data [30-32]. This methodological innovation is uniquely suited to address the limitations of traditional approaches: by capturing nonlinear associations and directional pathways, Bayesian networks enable us to precisely map how psychosocial factors interact through distinct routes in male and female populations and how these interactions ultimately contribute to depression and anxiety.

This study employed cross-sectional network analysis and Bayesian network analysis to investigate the structural relationships between psychological symptoms and psychosocial factors in a large sample of adults. Specifically, we aimed to (1) identify key nodes within the psychological symptom network, determining which factors exert the strongest influence to assess network stability and predictability, ensuring robustness and reliability; (2) explore potential causal pathways linking CT, ER, SES, and SS to mental health outcomes; and (3) examine gender differences in network structures, determining whether the relationships among these factors differ across male and female populations. By adopting a data-driven network modeling approach, this study seeks to enhance our understanding of the complex interconnections underlying mental health symptoms. More importantly, elucidating key intervention nodes within gender-specific networks could help inform targeted prevention and intervention strategies, ultimately contributing to the development of more effective, gender-sensitive mental health policies and interventions.

## Methods

### Participants

Participants were recruited from the Jingdong (JD) Insights Platform [33], an online survey platform commonly used for large-scale research in China. The platform provides access to a broad and diverse sample of participants from the general population. We distributed and collected online questionnaires across residential communities throughout China between May 1, 2022, and May 30, 2022. The questionnaire assessed sociodemographic characteristics, anxiety, depression, somatic symptoms, insomnia, CT, SES, ER, and SS. The inclusion criteria for this study were as follows: (1) age 18 years or older; (2) internet access via a smartphone or computer and independent completion of the questionnaires; (3) normal vision and hearing; and (4) must read and agree to the study information and consent form before beginning the survey. The exclusion criteria were as follows: (1) severe or unstable physical diseases; (2) known or self-reported cognitive impairment; (3) currently undergoing psychiatric medication or psychological interventions; and (4) clearly careless responses. We conducted a rigorous screening and cleaning process for the collected questionnaire data. First, participants who did not meet the inclusion or exclusion criteria were excluded (n=207). Then, responses deemed to be of low quality or invalid were removed (n=206), including straight-line answering, inconsistent or contradictory responses, extremely short completion times, and extreme or implausible answers.

### Measures

#### Depression, Anxiety, Insomnia, and Somatic Symptoms

Depression and anxiety symptoms over the past 2 weeks were assessed using the Patient Health Questionnaire-9 (PHQ9) and the Generalized Anxiety Disorder-7 (GAD7), respectively. Based on the severity, the symptoms were ranked as follows: normal, mild, moderate, moderate-severe, and severe. Insomnia symptoms over the past 4 weeks were evaluated with the Insomnia Severity Index (ISI), while somatic symptoms were measured using the Patient Health Questionnaire-15 (PHQ15), with higher scores indicating greater symptom severity. All the above scales have been validated to demonstrate satisfactory reliability in the Chinese population [34-37].

#### Childhood Trauma, Emotion Regulation, Self-Esteem, and Social Support

The childhood trauma questionnaire (CTQ) was used for self-assessment of 5 types of trauma experienced before the age of 16 years, including EA, physical abuse (PA), sexual abuse (SA), emotional neglect (EN), and physical neglect (PN) [38]. It consists of 28 items rated on a 5-point scale (1=never true to 5=very often true), with scores for each trauma type ranging from 5 to 25 and higher scores indicating greater severity. The emotional regulation questionnaire, which includes 2 dimensions of cognitive reappraisal (CR) and expressive suppression (ES), was used to assess regulation strategies in response to stressful events [39]. The total scores of CR and ES were calculated separately, with higher scores indicating greater use of the respective strategy to regulate emotions. The SES scale consists of 10 items, with higher scores indicating a higher level of self-worth and self-acceptance [40]. The SS revalued scale, comprising 3 dimensions, namely subjective support (SBS), objective support (OBS), and support utilization (SU), was used to assess the individuals’ SS condition [41]. Total and subscale scores were calculated by summing item responses, with higher scores indicating greater perceived support. These scales have demonstrated satisfactory validity across populations and are widely used in psychological research [41-44].

### Statistical Analyses

Network analyses were conducted using R (version 4.1.1), while regression analyses were conducted using SPSS (version 25.0). Categorical variables were presented as frequencies and percentages, whereas continuous variables were expressed as medians with interquartile ranges (IQRs). A *P* value of less than .05 was considered statistically significant.

#### Cross-Sectional Networks

##### Estimate Networks

The EBICglasso is a regularization method commonly used in psychology and neuroscience research to estimate sparse and interpretable networks [29,45,46]. To estimate a sparse network of relationships among depression, anxiety, insomnia, somatic symptoms, ER, SES, SS, and CT, we used the EstimateNetwork function in bootnet (version 1.6) package with EBICglasso regularization [45]. The threshold Boolean parameter of the EstimateNetwork function was set to TRUE. In the resulting networks, each node represents a distinct symptom, and the thickness of the edges between nodes indicates the strength of the association. Blue edges denote positive correlations, whereas red edges represent negative correlations.

##### Centrality Measures

In network analyses, 3 centrality measures, strength, closeness, and betweenness, are used to assess the importance of nodes within a network, each reflecting the role of a node in the network structure from a different perspective [47,48]. Strength reflects the total strength of direct connections between a node and other nodes, measuring the node’s direct influence. Closeness represents the average distance from a node to all other nodes, reflecting its accessibility and global importance within the network. Betweenness refers to the frequency with which a node appears on the shortest paths between other node pairs, indicating its bridging role. R package qgraph (version 1.9.8) was used to compute the centrality measures of different symptoms nodes and draw a centrality plot [49].

##### Network Accuracy and Stability

Network accuracy and stability were assessed by performing 1000 bootstrap samples and bootstrap difference tests on the data using the bootnet function from the bootnet (version 1.6) package [45]. The stability of the network between nodes and edges was estimated using the Correlation Stability Coefficient (CS-C) index, with the coefficient size directly reflecting network stability. For a network to be considered stable, the CS-C should not be lower than 0.25 [45]. In addition, network accuracy was evaluated by: (1) the bootstrapped 95% CIs for the edge weights and (2) the differences between nodes and between edges. The combination of these results effectively provides a comprehensive evaluation of the network’s accuracy and stability.

##### Network Predictability

Network predictability refers to the extent to which the changes in a specific node within the network can be predicted by changes in the nodes to which it is connected [50]. We used the mgm function from the mgm (version 1.2‐14) package to obtain the Mixed Graphical Models (MGMs), with the regularization parameter for the model set to EBIC [50]. The predict function was then used to generate the model’s predictability values, which were evaluated and interpreted using *R*^2^. An *R*^2^ value of 0 indicates that the current node is not explained by any of the other nodes in the network, while an *R*^2^ value of 1 represents perfect prediction. Attentionally, due to the influence of multiple factors (both modeled and unmodeled), *R*^2^ should be considered only as a reference measure.

##### Network Comparisons

The network comparison test (NCT) was used to evaluate whether significant differences existed between the structures of pairwise networks [51]. The NCT function from the NCT (version 2.2.2) package was applied to perform 1000 random permutation tests with Bonferroni–Holm correction, yielding the Spearman correlations (*r_s_*) and *P* values for pairwise network comparisons across the following parameters: global strength invariance, network structure invariance, edge weights similarity, strength similarity, and predictability similarity [51]. The *r_s_* range from 0 to 1, with values closer to 1 indicating a higher similarity between the two networks.

### Bayesian Networks

#### Directed Acyclic Graphs

Directed Acyclic Graph (DAG) is a model in Bayesian network that represents the underlying potential causal relationships among symptoms [30]. Given that DAG structures are sensitive to confounding factors, we incorporated 9 additional potential confounders beyond the symptoms included in the previous network analyses. These variables included education level, marital status, income, children, living status, and others. This adjustment aimed to improve the accuracy and causal interpretability of the DAG results. First, the structure of the DAG was constructed using 2000 bootstrap samples via the lapply function. Bayesian network structure learning was then performed for each sample using the hill-climbing algorithm from the bnlearn (version 5.0.1) package, with parallel computing efficiency enhanced through the pbapply (version 1.7‐2) package [52]. Data were used without standardization as the hill-climbing algorithm with aic-cg does not depend on the variable scale. We incorporated blacklists based on prior knowledge from experts and the literature. The blacklists in this study include the following: (1) arcs that violate known conventional directional relationships; (2) restricting 5 indicators (gender, education level, marital status, children, and history of mental illness) to be used only as parent nodes; (3) restricting PHQ9 and GAD7 to be used only as child nodes (where node A points to node B, making A the parent and B the child). Second, to better interpret the network, the frequency of each edge in the Bayesian network structures learned from the 2000 bootstrap samples was calculated. Edges with a frequency greater than 90% were retained as the edges of the output network structure, which was then exported using the averaged.network function.

#### Calculation of Edge Coefficients via Regression Analyses

The weights of edges in the DAG are generally represented by the strength or frequency of the relationships between nodes, making it difficult to directly express the correlation coefficients and the direction of correlation (positive or negative) between nodes. To further quantify these associations and provide a more interpretable estimation of effect sizes between nodes in the DAG, we conducted regression analyses on the edges between nodes based on the obtained DAG structure, replacing traditional edge weights with regression coefficients [53]. The process is as follows: First, the parent–child relationships obtained from the hill-climbing algorithm were extracted. Then, multiple regression analysis was performed by using the child node as the dependent variable, with the parent nodes associated with the child node as independent variables, to obtain the regression coefficients and *P* values. Finally, the regression coefficients obtained were used to represent the thickness of the edges in the DAG, with edge direction indicated by color: blue edges represent positive correlations, and red edges indicate negative correlations. This method of representing edge weights through regression coefficients provides a better visualization and interpretation of the strength and direction (positive or negative) of the relationships between nodes in the DAG.

### Sample Size

According to established sample size guidelines and previous studies in network analysis [45,54,55], a participant-to-parameter ratio greater than 10:1 is recommended to achieve stable network estimation. The network in this study comprised 15 nodes and 105 possible edges, requiring more than 1050 participants in both the male and female subsamples to meet this criterion. Our total sample size was 6105 participants (3648 male participants and 2457 female participants), which satisfies this requirement.

### Ethical Considerations

This study was approved by the ethics committee of Tongji Hospital Affiliated Tongji Medical College, Huazhong University of Science and Technology (approval number: TJ-IRB20220408) and was registered in the Chinese Clinical Trial Registry (registration number: ChiCTR2200059155). Online informed consent was obtained from all participants before data collection. All responses were collected anonymously, and participants’ personal information was securely stored and kept confidential in accordance with relevant data protection regulations. Each participant who fully completed the questionnaire received Jingdong coupons as a reward. Additionally, the manuscript and supplementary materials contain no identifiable personal information, including images, videos, or location data.

## Results

### Participant Characteristics

**Figure 1.**
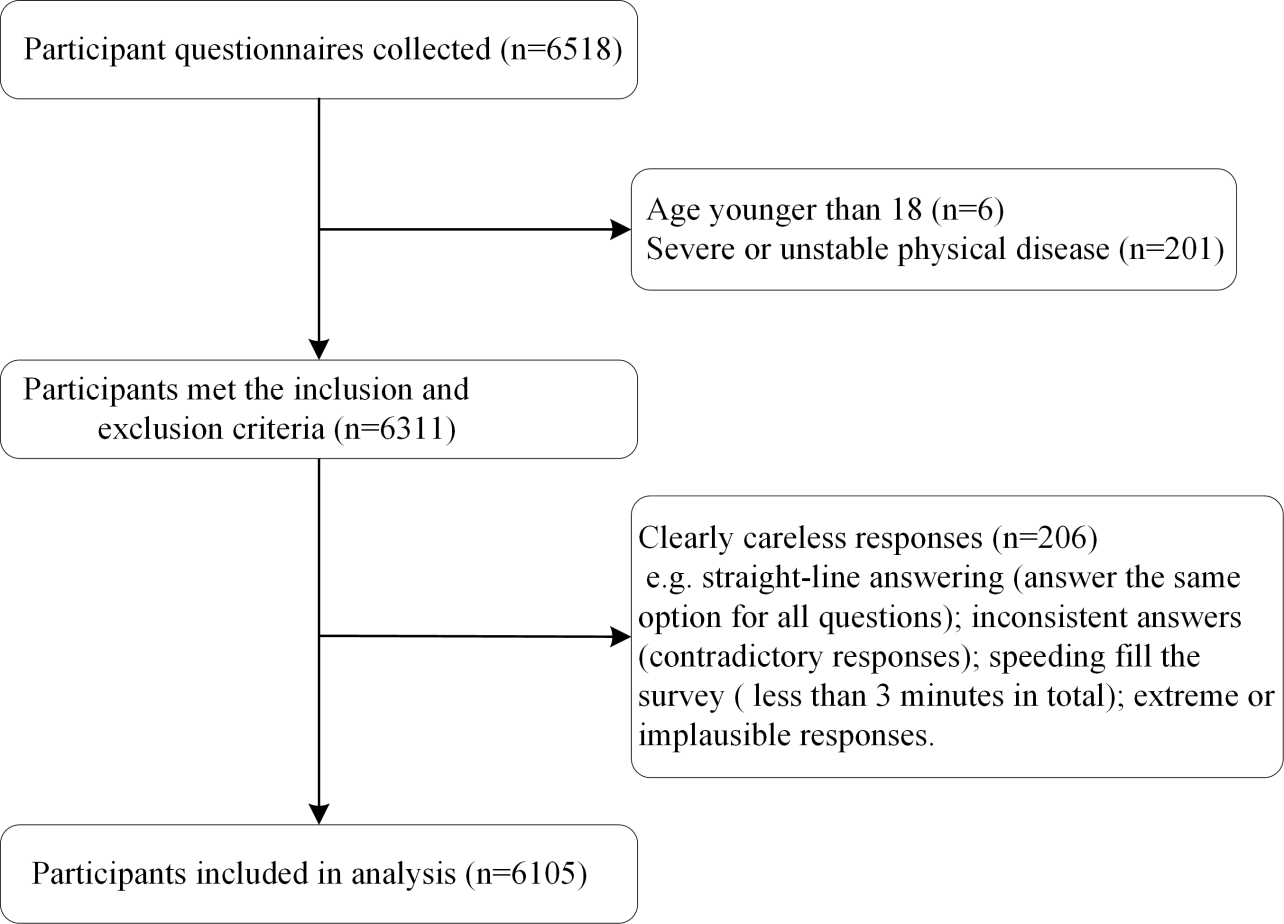
Flowchart of participant data selection.

A total of 6105 participants were included in the final analysis (Figure 1), of whom 3648 (59.75%) were male and 2457 (40.25%) were female (Table 1). Among the participants, 64.13% were aged approximately 31–54 years, and 72.20% had received education at the undergraduate or junior college level. Table S1 in Multimedia Appendix 1 provides a detailed presentation of the demographic characteristics and scale scores of the included participants. In the current cohort, the prevalence of moderate or more severe depression was 12.06% in the overall sample, 13.00% in male participants, and 10.66% in female participants. Similarly, the prevalence of moderate or more severe anxiety in the overall sample, male participants, and female participants was 7.93%, 8.58%, and 6.96%, respectively (Table S2 in Multimedia Appendix 1).

**Table 1.** Demographic characteristics and scale scores of participants.

Characteristic	All (N=6105), n (100%)	Male (n=3648), n (59.75%)	Female (n=2457), n (40.25%)
Age (years)			
18–30	2040 (33.42)	1159 (31.77)	881 (35.89)
31–54	3915 (64.13)	2398 (65.73)	1517 (61.74)
55–80	150 (2.46)	91 (2.49)	59 (2.40)
Educational level			
Junior high school or below	103 (1.69)	77 (2.11)	26 (1.06)
High school or technical secondary school	513 (8.40)	334 (9.16)	179 (7.29)
Undergraduate or junior college	4408 (72.20)	2600 (71.27)	1808 (73.59)
Master’s or above	1081 (17.71)	637 (17.46)	444 (18.07)
Marital status			
Unmarried	1624 (26.60)	1038 (28.45)	586 (23.85)
Married	4287 (70.22)	2500 (68.53)	1787 (72.73)
Divorced or widowed	194 (3.18)	110 (3.02)	84 (3.42)
Children			
No children	2309 (37.82)	1464 (40.13)	845 (34.39)
One child	2837 (46.47)	1620 (44.41)	1217 (49.53)
More than one child	959 (15.71)	564 (15.46)	395 (16.08)
Household income			
Less than 2000 RMB/month (280.52 $/month)	109 (1.79)	68 (1.86)	41 (1.67)
2000~5000 RMB/month (280.52~701.30 $/month )	504 (8.26)	272 (7.46)	232 (9.44)
5000~10000 RMB/month (701.30~1402.60 $/month )	1706 (27.94)	1027 (28.15)	679 (27.64)
10000~20000 RMB/month (1402.60~2805.17 $/month)	2209 (36.18)	1328 (36.40)	881 (35.86)
More than 20000 RMB/month (2805.17 $/month )	1577 (25.83)	953 (26.12)	624 (25.40)
Living status			
Living alone	1116 (18.28)	789 (21.63)	327 (13.31)
Living with family members	4921 (80.61)	2823 (77.38)	2098 (85.39)
Living with others	68 (1.11)	36 (0.99)	32 (1.30)
Drinking			
No	2889 (47.32)	1287 (35.28)	1602 (65.20)
Light	3147 (51.55)	2298 (62.99)	849 (34.55)
Heavy (≥50 g/day)	69 (1.13)	63 (1.73)	6 (0.24)
Smoking			
No	5206 (85.27)	2801 (76.78)	2405 (97.88)
Yes	899 (14.73)	847 (23.22)	52 (2.12)
History of mental illness			
No	6047 (99.05)	3626 (99.40)	2421 (98.53)
Yes	58 (0.95)	22 (0.60)	36 (1.47)
Drug abuse			
No	6094 (99.82)	3642 (99.84)	2452 (99.80)
Yes	11 (0.18)	6 (0.16)	5 (0.20)
PHQ9^a^^,^^b^	3.00 (7.00)	3.00 (7.00)	3.00 (7.00)
GAD7^b^^,^^c^	1.00 (6.00)	1.00 (6.00)	2.00 (6.00)
ISI^b^^,^^d^	3.00 (7.00)	3.00 (7.00)	2.00 (6.00)
PHQ15^b^^,^^e^	2.00 (6.00)	2.00 (5.00)	3.00 (6.00)
ER^b^^,^^f^			
CR^g^	27.00 (7.00)	26.00 (7.00)	28.00 (8.00)
ES^h^	15.00 (6.00)	16.00 (5.00)	14.00 (6.00)
SES^b^^,^^i^	30.00 (8.00)	30.00 (9.00)	30.00 (7.00)
SSRS^b^^,^^j^			
OBS^k^	8.00 (5.00)	8.00 (5.00)	8.00 (4.00)
SBS^l^	22.00 (9.00)	22.00 (9.00)	22.00 (9.00)
SU^m^	7.00 (2.00)	6.00 (3.00)	7.00 (2.00)
CTQ^b^^,^^n^			
EA^o^	6.00 (2.00)	6.00 (2.00)	6.00 (2.00)
PA^p^	5.00 (1.00)	5.00 (1.00)	5.00 (0.00)
SA^q^	5.00 (0.00)	5.00 (0.00)	5.00 (0.00)
EN^r^	10.00 (7.00)	11.00 (7.00)	10.00 (7.00)
PN^s^	8.00 (5.00)	8.00 (6.00)	7.00 (5.00)

a PHQ9: Patient Health Questionnaire-9.

b Medians (IQRs).

c GAD7: Generalized Anxiety Disorder-7.

d ISI: Insomnia Severity Index.

e PHQ15: Patient Health Questionnaire-15.

f ER: emotion regulation.

g CR: cognitive reappraisal.

h ES: expressive suppression.

i SES: self-esteem

j SSRS: Social Support Revalued Scale.

k OBS: objective support.

l SBS: subjective support.

m SU: support utilization.

n CTQ: Childhood Trauma Questionnaire.

o EA: emotional abuse.

p PA: physical abuse.

q SA: sexual abuse.

r EN: emotional neglect.

s PN: physical neglect.

### Network of EBICglasso

**Figure 2.**
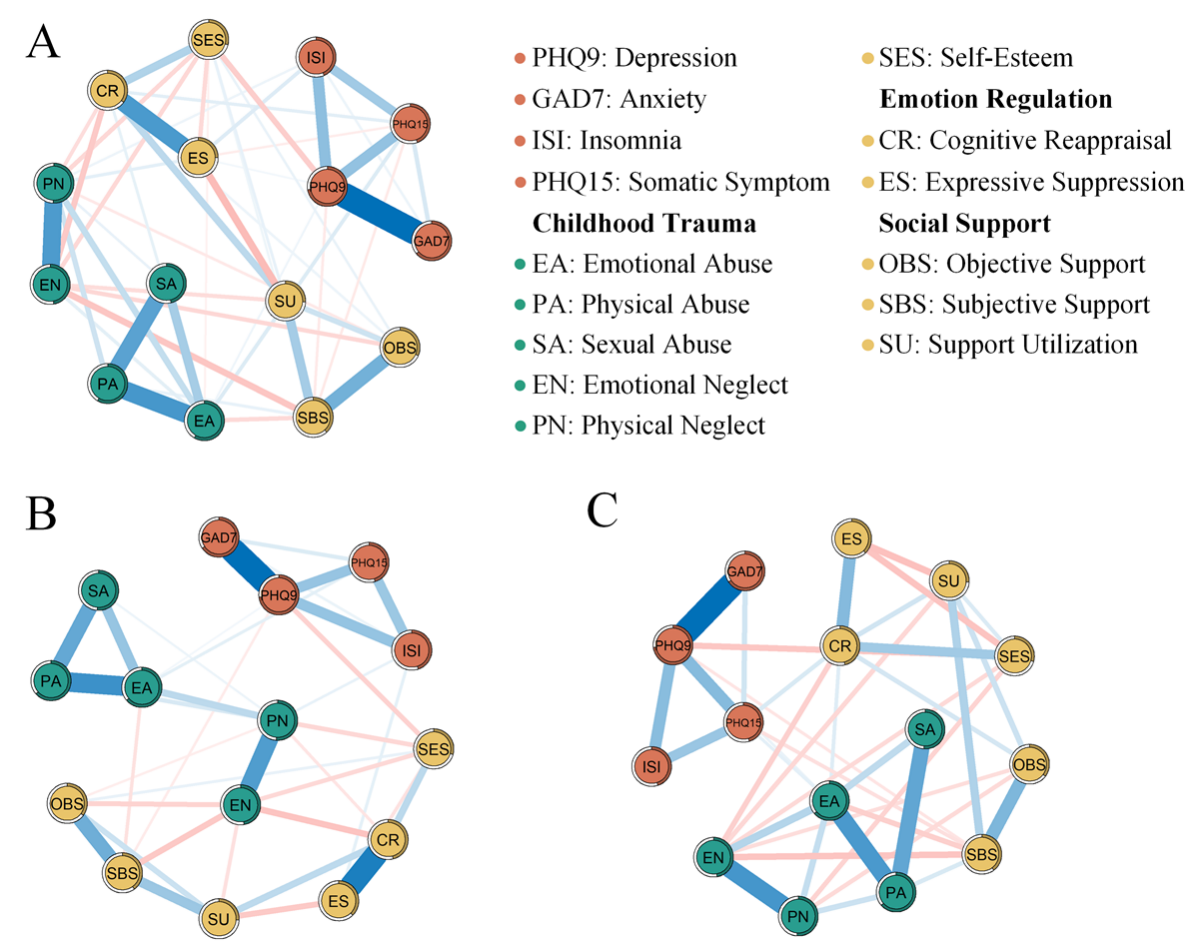
Cross-sectional network structures among all participants, male participants, and female participants. The half-circle of each node represents the node’s predictability (*R*^2^). The blue and red edges indicate positive and negative correlations, respectively, with thicker edges indicating stronger associations. (A) All participants; (B) Male participants; and (C) Female participants.

Figure 2 shows the estimated network structures for the overall sample, male participants, and female participants. The network included a total of 15 nodes, with a maximum of 105 possible edges. In the overall sample, 50 edges were observed (34 positive and 16 negative correlations), accounting for 47.62% of the possible edges. Among male participants, 37 edges were present (24 positive and 13 negative correlations), representing 35.24% of the possible edges. For female participants, 38 edges were present (25 positive and 13 negative correlations), accounting for 36.19% of the possible edges. Notably, the overall network contained more edges, indicating a more complex structure. The 3 network analyses revealed strong associations among PHQ9, GAD7, ISI, and PHQ15, with a more intricate structure in women compared with that in men. Across all networks, emotional neglect (EN) negatively correlated with cognitive reappraisal (CR), SS, and SES, suggesting its widespread impact on psychological functioning. Gender differences emerged in key edges, with PN negatively linked to CR in male participants, while the ISI correlated positively with PN and ES, indicating associations between childhood neglect, sleep disturbances, and expressive suppression. Men also exhibited a stronger CR-ES connection, reflecting distinct ER patterns. In contrast, women showed a positive correlation between CR and OBS, as well as PHQ15, while SBS was negatively associated with PHQ15, suggesting better SS was linked to fewer somatic symptoms. Additionally, SU was positively associated with SES, highlighting SS as a crucial factor in SES for women (Table S3-5 in Multimedia Appendix 1).

**Figure 3.**
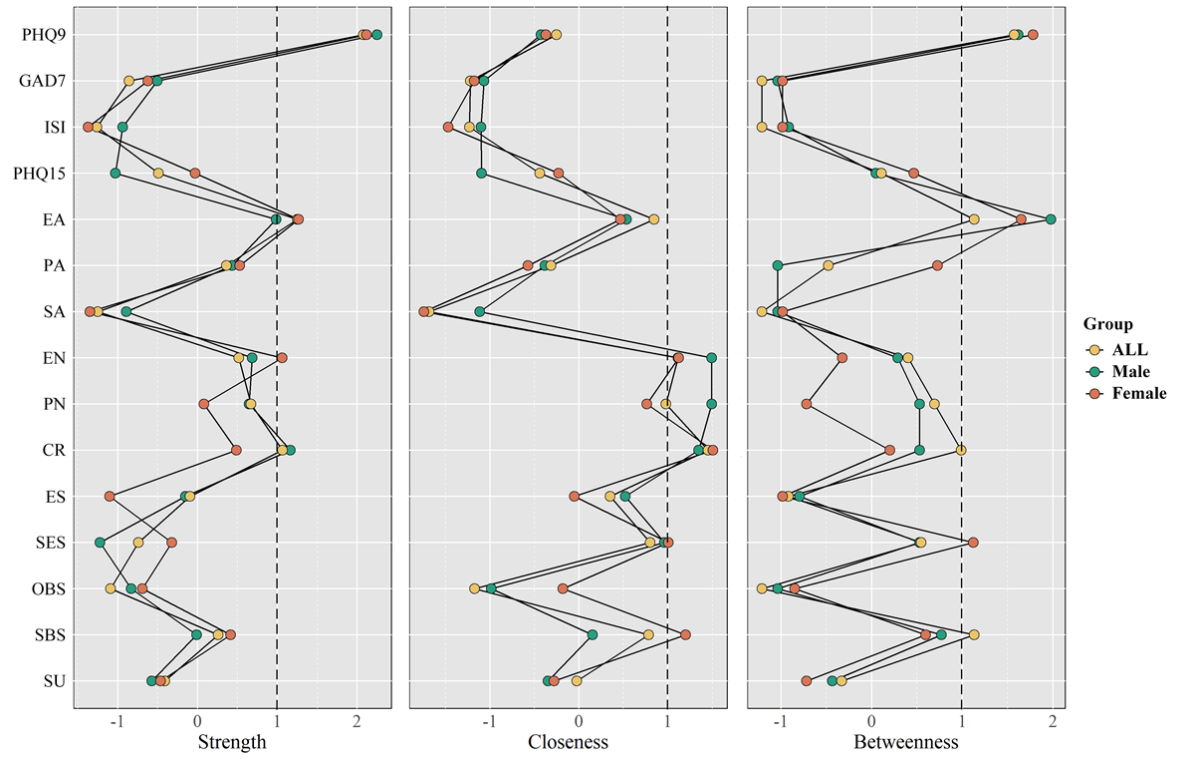
Node centrality measures of strength, closeness, and betweenness in the 3 cross-sectional networks. PHQ9, Patient Health Questionnaire-9; GAD7, Generalized Anxiety Disorder-7; ISI, Insomnia Severity Index; PHQ15, Patient Health Questionnaire-15; EA, emotional abuse; PA, physical abuse; SA, sexual abuse; EN, emotional neglect; PN, physical neglect; CR, cognitive reappraisal; ES, expressive suppression; SES, self-esteem; OBS, objective support; SBS, subjective support; SU, support utilization.

The standardized centrality measures of strength, closeness, and betweenness are plotted in Figure 3. In the male population, PHQ9 (2.25) and CR (1.16) demonstrated the highest strength centrality, EN (1.50), PN (1.49), and CR (1.35) showed the highest closeness centrality, and PHQ9 (1.62) and EA (1.98) exhibited the highest betweenness centrality. In the female population, PHQ9 (2.12), EA (1.26), and EN (1.12) displayed the highest strength centrality, EN (1.12), CR (1.51), and SBS (1.20) showed the highest closeness centrality, and PHQ9 (1.78), EA (1.65), and SES (1.12) exhibited the highest betweenness centrality (Table S6 in Multimedia Appendix 1).

We conducted 1000 bootstrap sampling and bootstrap difference tests on the data to test the stability of the three networks, as shown in Figure S1 in Multimedia Appendix 1. Strength centrality exhibited the highest stability across all networks (CS-C=0.75), indicating strong reliability. Betweenness centrality demonstrated the lowest stability but remained within the acceptable threshold (Table S7 in Multimedia Appendix 1). Furthermore, the bootstrapped 95% CIs for the edge weights and the differences between nodes and edges exhibited the accuracy of the networks, as shown in Figure S2-4 in Multimedia Appendix 1. While the overall network predictability was moderate but acceptable (44.7%), core psychological symptoms such as depression (72.4%) and anxiety (64%) exhibited strong predictability, supporting the robustness of the model (Table S8 in Multimedia Appendix 1).

The network comparison test (NCT) with Holm–Bonferroni correction assessed differences across the overall, male, and female networks. As shown in Table 2, the male and female networks exhibited high similarity in edge weights (*r*_s_=0.941, *P*<.010), strength centrality (*r*_s_=0.844, *P*<.010), and predictability (*r*_s_=0.850, *P*<.010), indicating largely comparable network structures. However, the network structure invariance test was significant (*P*=.001), suggesting notable gender differences in specific network connections. In contrast, the global strength invariance test was nonsignificant (*P*=.558), indicating similar overall connectivity strength between male and female participants. These findings suggest that while the general structure of the networks was similar across genders, the specific relationships among psychological factors vary, highlighting potential gender-specific patterns in mental health symptom interactions.

**Table 2.** Internetwork similarity indexes and network comparison tests.^a^

Comparison	Edge weights similarity	Strength similarity	Predictability similarity	Global strength invariance	Network structure invariance
ALL-male	*r*_s_=0.984^b^	*r*_s_=0.957^b^	*r*_s_=0.955^b^	*P*=.113	*P*=.002
ALL-female	*r*_s_=0.973^b^	*r*_s_=0.905^b^	*r*_s_=0.895^b^	*P*=.109	*P*=.003
Male-female	*r*_s_=0.941^b^	*r*_s_=0.844^b^	*r*_s_=0.850^b^	*P*=.558	*P*=.001

a Spearman correlations (*r*_s_) were computed; *P* values were adjusted for Holm–Bonferroni correction.

b *P*<.010.

### Bayesian Networks

In the Bayesian network analyses, 9 confounding factors were included: education levels, marital status, children, income, living status, drinking, smoking, history of mental illness, and drug abuse, considering that these factors may also influence depression and anxiety in the population. To obtain a clear and focused DAG network diagram, we used the hill-climbing algorithm and retained high-frequency edges as the edges of the output network structure. Regression analyses were then performed based on the resulting DAG structure to calculate the edge coefficients.

**Figure 4.**
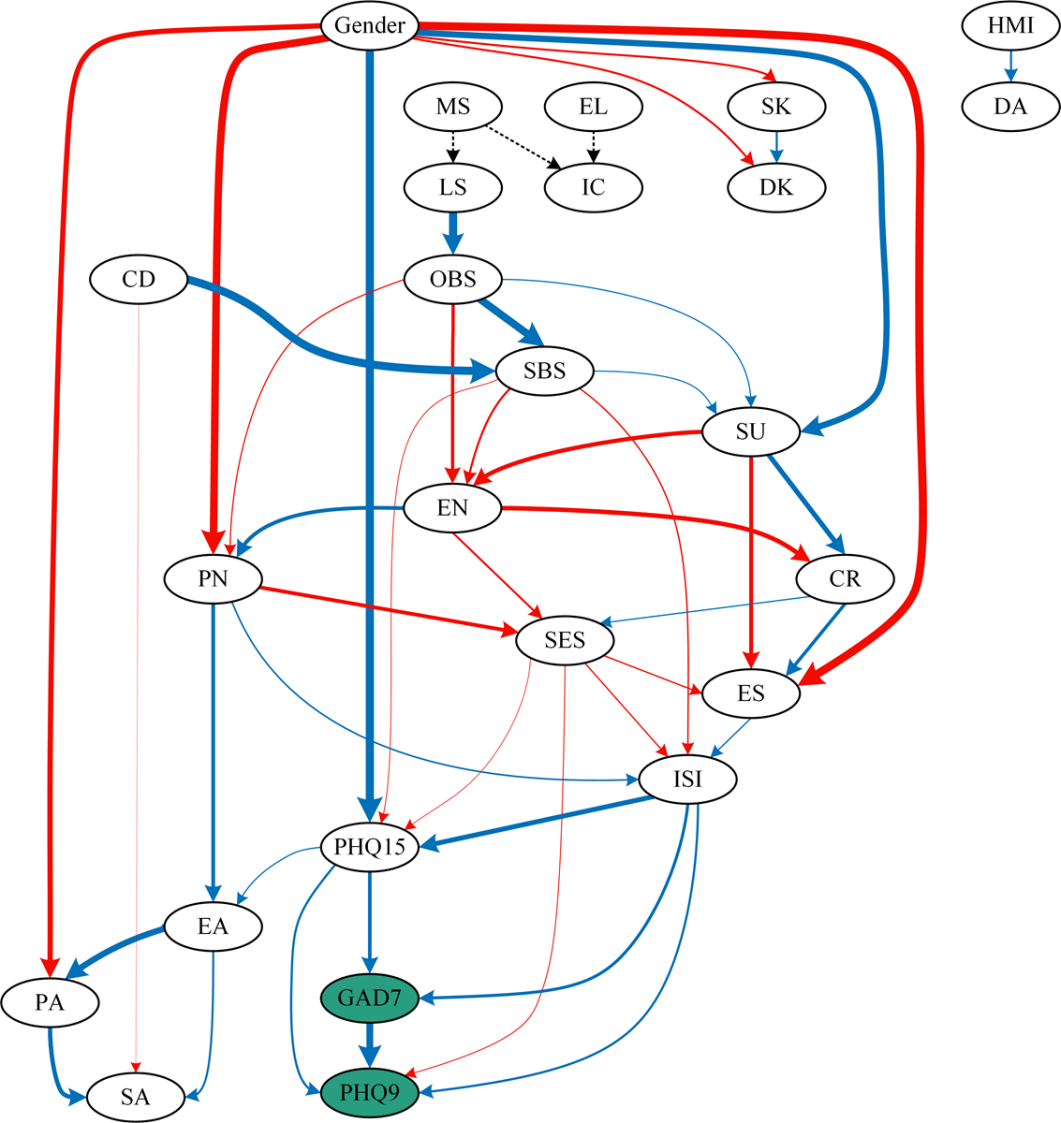
DAG of all participants. Blue edges represent positive correlations; red edges represent negative correlations; dashed edges indicate correlations with categorical variables (directionality undetermined); thicker edges indicate higher edge coefficients. Depression (PHQ9) and anxiety (GAD7) are highlighted in green ellipses. In the classification of gender, male gender was used as the reference category. PHQ9, Patient Health Questionnaire-9; GAD7, Generalized Anxiety Disorder-7; ISI, Insomnia Severity Index; PHQ15, Patient Health Questionnaire-15; EA, emotional abuse; PA, physical abuse; SA, sexual abuse; EN, emotional neglect; PN, physical neglect; CR, cognitive reappraisal; ES, expressive suppression; SES, self-esteem; OBS, objective support; SBS, subjective support; SU, support utilization; CD, children; MS, marital status; LS, living status; EL, education level; IC, income; SK, smoking; DK, drinking; HMI, history of mental illness; DA, drug abuse.

Figure 4 presents the overall DAG network, and the frequency of network edges is presented in Table S9 in Multimedia Appendix 1. It shows that gender was the most influential upstream node, triggering key psychological variables such as PN, PA, SU, ES, and PHQ15, which subsequently affected depression (PHQ9) and anxiety (GAD7). Living status (LS) influenced downstream symptoms through social support (OBS), while CT (EN, PN) played a significant role in shaping mental health outcomes. However, PA, EA, and SA did not exhibit direct effects on depression or anxiety, suggesting a more indirect influence through other mediators. SES was an important node in the midstream and a bridge in the whole network, which was regulated by nodes above, thus affecting nodes downstream. Three major potential causal pathways were identified: (1) gender → PN / SS / expressive suppression→ SES → insomnia / somatization → anxiety → depression; (2) gender → somatization → anxiety → depression; and (3) living status → SS → EN → SES → insomnia / somatization → anxiety → depression.

**Figure 5.**
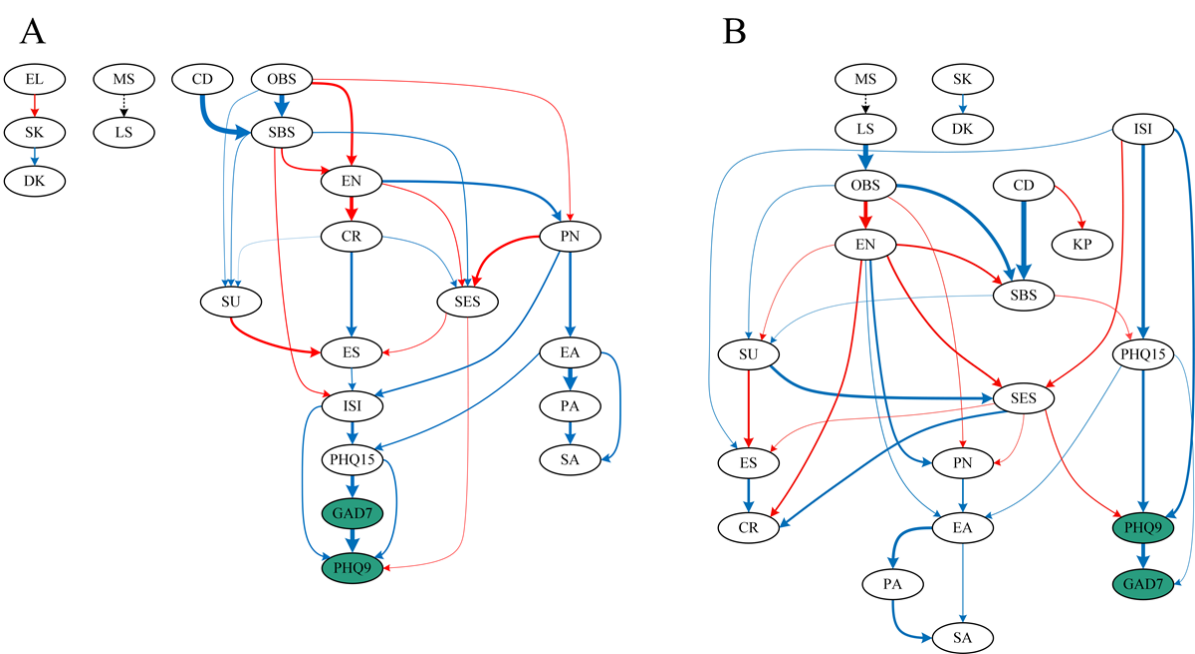
Directed acyclic graphs (DAGs) of males and females. Blue edges represent positive correlations; red edges represent negative correlations; dashed edges indicate correlations with categorical variables (directionality undetermined); thicker edges indicate higher edge coefficients. Depression (PHQ9) and anxiety (GAD7) are highlighted in green ellipses. (A) Male participants; (B) Female participants. PHQ9, Patient Health Questionnaire-9; GAD7, Generalized Anxiety Disorder-7; ISI, Insomnia Severity Index; PHQ15, Patient Health Questionnaire-15; EA, emotional abuse; PA, physical abuse; SA, sexual abuse; EN, emotional neglect; PN, physical neglect; CR, cognitive reappraisal; ES, expressive suppression; SES, self-esteem; OBS, objective support; SBS, subjective support; SU, support utilization; CD, children; MS, marital status; LS, living status; EL, education level; IC, income; SK, smoking; DK, drinking.

Given the significant role of gender, separate DAG networks for male and female participants were analyzed (Figure 5; Table S10-11 in Multimedia Appendix 1). In the male network, the most upstream nodes were OBS and children, influencing SU, SBS, EN, and PN, which subsequently affected anxiety and depression. CR, ES, and PN emerged as key midstream nodes, serving as bridges between early experiences and symptom development. In male participants, GAD7 was the parent node of PHQ9, suggesting anxiety precedes depression. In female participants, the most upstream nodes were LS, children, and ISI, with the ISI showing a stronger impact on anxiety and depression than in male participants. Unlike men, CR, ES, and PN did not play a central bridging role in the female network. Instead, SES was a key mediator linking SS and CT to depression and anxiety. Interestingly, PHQ9 was the parent node of GAD7 in female participants, indicating depression precedes anxiety, a pattern opposite to that observed in male participants. The main pathway of influence in male participants was as follows: SS → EN / PN → SES / ER → insomnia → somatization → anxiety → depression. There were two main influence pathways in female participants: living status / children→ SS → EN→ SES → depression → anxiety; insomnia → somatization → depression → anxiety.

## Discussion

### Principal Results

This study aimed to investigate the complex interplay between depression, anxiety, CT, SES, SS, emotional regulation, and potential confounders in a large cohort, with a particular focus on gender differences. To achieve this, we simultaneously applied 2 network analysis techniques: cross-sectional network analysis and Bayesian network analysis. Cross-sectional network analysis emphasizes the correlation and strength of connections between psychosocial factors and symptoms, while the Bayesian network analysis represents conditional dependencies and potential causal relationships with incorporating key sociodemographic confounders to better reflect directional influences. Together, these complementary methods enabled a comprehensive understanding of the associative and causal structures underlying mental health symptoms and allowed us to identify gender-specific pathways that may inform targeted interventions for depression and anxiety.

The cross-sectional network revealed that while the overall network structures were largely similar across genders, significant differences emerged in specific connections. This indicates that, although the general association patterns among symptoms were comparable between male and female participants, certain interrelationships were more pronounced or uniquely manifested depending on gender. A key finding in the Bayesian network was that anxiety precedes depression in men, whereas depression precedes anxiety in women, reinforcing prior evidence of gender differences in symptom progression [20,56-58]. This aligns with theories suggesting that men are more likely to externalize stress responses, leading to anxiety-driven symptom trajectories, whereas women’s greater tendency for rumination and emotional distress fosters depression first, later triggering anxiety [25,59-61]. Clinically, these findings emphasize that early intervention for anxiety in men may help prevent the onset of depression, while targeting depression in women may reduce secondary anxiety symptoms.

Our study, using DAGs, provides a refined understanding of gender-specific psychosocial mechanisms influencing mental health. In female participants, SES and SS, particularly SBS and support utilization (SU), played a central role, whereas in male participants, ER strategies, including cognitive reappraisal (CR) and expressive suppression (ES), were more influential. Additionally, in the cross-sectional network analysis, ER exhibited higher standardized centrality indices in male participants, whereas SES and SBS showed higher standardized centrality indices in female participants. These findings build upon prior research by offering a more structured potential causal framework [23,27,62], reinforcing that women’s mental health is more closely linked to self-worth and interpersonal support, while men rely more on internal ER strategies. This refined understanding underscores the need for tailored interventions, emphasizing SES and SS enhancement for women and ER training for men. By systematically delineating the interplay between these factors, our study advances the field by identifying gender-specific protective and risk factors with greater clarity.

CT is a well-established risk factor for mental health disorders, with previous studies linking EN, PN, PA, EA, and SA to increased vulnerability to anxiety and depression in adulthood [10,58,63]. In this study, we observed a significant correlation between EN and factors such as CR, SS, and SES, highlighting its broad influence on psychological functioning in both male and female networks. However, both overall and gender-specific DAG analyses indicated that CT did not exert a direct effect on anxiety or depression. Instead, its impact was mediated through psychosocial factors such as SS, SES, and ER, supporting the notion that CT influences mental health primarily through complex indirect pathways rather than serving as a direct predictor of symptom severity. Moreover, gender differences emerged in the mediating pathways of CT. In male participants, childhood neglect (PN, EN) primarily impacted ER (CR, ES), contributing to insomnia, somatic symptoms, and increased anxiety and depression risk. In female participants, childhood trauma mainly influenced SES and SS, which directly affected anxiety and depression. These findings highlight the need for gender-specific interventions, focusing on SES and SS in females and improving ER in males to mitigate the long-term mental health effects of CT.

Additionally, insomnia (ISI) was identified as a key upstream node in the female network, exerting a significant impact on both anxiety and depression. This aligns with previous research showing that women are more susceptible to sleep disturbances [64,65], which in turn exacerbate emotional distress. These findings suggest that sleep interventions may be particularly beneficial for preventing depressive symptoms in females.

Despite these gender differences, several important consistencies emerged across networks. First, the strong associations among depression (PHQ9), anxiety (GAD7), insomnia (ISI), and somatic symptoms (PHQ15) were stable across genders, suggesting a robust symptom cluster underlying emotional distress. Second, as previously noted, EN consistently negatively correlated with CR, SS, and SES in both male and female networks. This indicates that insufficient emotional responsiveness in childhood may undermine ER capacities and interpersonal trust, thereby increasing vulnerability to psychopathology in adulthood, regardless of gender. Moreover, the Bayesian network analysis revealed that the effects of CT on depression and anxiety were primarily mediated by intermediate psychological factors rather than acting directly. This suggests a shared pathological pathway, CT → psychological impairment → symptom onset, that may operate similarly in both sexes, with differences arising mainly in the specific mediating variables involved.

The strengths of this study are as follows: primarily, this study provides novel insights into gender-specific depression and anxiety pathways through a large-scale sample and two complementary network analyses. This approach enables a more reliable and stable exploration of complex network relationships. Second, by integrating Bayesian networks with regression, this study moves beyond association-based models, enabling potential causal inference and effect size estimation for a clearer understanding of symptom interactions. Third, unlike previous research focused solely on depression-anxiety links, this study incorporates CT, SES, SS, and ER, uncovering their distinct mediating roles across genders. Fourth, this is the first study to map gender differences in symptom networks, showing that anxiety drives depression in men, while depression drives anxiety in women. The findings highlight SES and social support as key for women and ER for men, offering data-driven targets for gender-specific interventions. By applying Bayesian causal inference, this study advances mental health research toward precision-based, personalized interventions.

### Limitations

However, there are several limitations that need to be considered. First, the cross-sectional design of this study limited the ability to draw causal conclusions. While Bayesian networks allow causal inference, they do not establish true temporal causality. The inferred pathways should be interpreted as potential causal directions rather than definitive evidence of causal sequences. Future research should incorporate longitudinal data to validate symptom progression pathway. Second, although our Bayesian network analysis controlled for several confounding factors, unmeasured variables, such as personality traits or environmental stressors, may also significantly influence the development of depression and anxiety. Third, this study was conducted in a nonclinical Chinese population, and findings may not generalize to other cultural contexts or clinical populations. In addition, the online survey format may introduce self-selection, recall, and reporting biases, with an overrepresentation of highly educated individuals, potentially limiting generalizability. Replication in diverse samples is necessary. Although the study included key psychosocial factors, future models could integrate neurobiological, genetic, and environmental stressors for a more comprehensive understanding of mental health disorders. Last, neither cross-sectional nor Bayesian causal networks capture higher-order interactions (eg, age×gender, income×gender), which may influence mental health outcomes. Approaches exploring these interactions could complement our findings and provide a fuller understanding of gender differences in mental health.

### Conclusions

This study offers novel insights into gender-specific symptom networks in depression and anxiety, emphasizing distinct pathways that may inform theory and practice. Rather than reiterating individual symptom targets, our findings highlight broader implications for personalized mental health care: early anxiety-focused interventions may be especially beneficial for men, while enhancing self-worth, sleep, and SS may be more effective for women. These results contribute to a gender-sensitive framework of affective disorders, supporting the development of targeted, data-informed strategies in both clinical and public health settings. Future research should prioritize longitudinal and cross-cultural validation, as well as integration of biological markers, to advance precision mental health.

## Supplementary material

10.2196/76913 Multimedia Appendix 1 Supplementary tables and figures detailing variables, network analyses, and node metrics in the network study.

10.2196/76913 Checklist 1 STROBE Checklist

## Abbreviations

CR: cognitive reappraisal
CS-C: correlation stability coefficient
CT: childhood trauma
CTQ: childhood trauma questionnaire
DA: drug abuse
DAG: directed acyclic graph
EA: emotional abuse
EBICglasso: extended Bayesian information criterion graphical LASSO
EL: education levels
EN: emotional neglect
ER: emotion regulation
ES: expressive suppression
GAD7: Generalized Anxiety Disorder-7
HMI: history of mental illness
HPA: hypothalamic-pituitary-adrenal
IQRs: interquartile ranges
ISI: Insomnia Severity Index
LS: living status
MGM: Mixed Graphical Models
NCT: Network Comparison Test
OBS: objective support
PA: physical abuse
PHQ15: Patient Health Questionnaire-15
PHQ9: Patient Health Questionnaire-9
PN: physical neglect
SA: sexual abuse
SBS: subjective support
SES: self-esteem
SS: social support
SU: support utilization
