# Supplementary material for: Gender Differences in Psychosocial Pathways to Depression and Anxiety: Cross-Sectional and Bayesian Causal Network Study
Source: J Med Internet Res. 2025 Oct 3;27:e76913. doi: 10.2196/76913 (PMC12494359; doi:10.2196/76913)
Supplement: Multimedia Appendix 1 [file jmir-v27-e76913-s001.docx]

**Table S1** The descriptions of the variables in the analysis.

| Abbreviation | Type | Description |
| --- | --- | --- |
| PHQ9 | Continuous | Patient Health Questionnaire 9. |
| GAD7 | Continuous | Generalized Anxiety Disorder 7. |
| ISI | Continuous | Insomnia Severity Index. |
| PHQ15 | Continuous | Patient Health Questionnaire 15. |
| CR | Continuous | Cognitive reappraisal. A type of emotion regulation strategy that refers to changing the way one thinks about the meaning of a stimulus. |
| ES | Continuous | Expressive suppression. A type of emotion regulation strategy that refers to the active inhibition of outward displays of subjective emotional experience. |
| SES | Continuous | Self-esteem. Evaluate feelings of self-worth and self-acceptance. |
| OBS | Continuous | Objective support. A dimension of the SSRS, referring to tangible, visible, or practical support, which includes both material direct support and the existence and participation in social networks and group relationships. |
| SBS | Continuous | Subjective support. A dimension of the SSRS, referring to the emotional experience of being respected, supported, and understood within society. |
| SU | Continuous | Support utilization. A dimension of the SSRS, referring to differences in the utilization of social support by individuals. |
| EA | Continuous | Emotional abuse. A dimension of the CTQ, referring to verbal or behavioral actions that cause emotional harm and diminish self-worth. |
| PA | Continuous | Physical abuse. A dimension of the CTQ, referring to the infliction of physical harm, such as hitting or violent acts. |
| SA | Continuous | Sexual abuse. A dimension of the CTQ, referring to coercion or forced participation in sexual activities. |
| EN | Continuous | Emotional neglect. A dimension of the CTQ, referring to the failure to provide adequate emotional support or care. |
| PN | Continuous | Physical neglect. A dimension of the CTQ, referring to the failure to provide basic physical needs, such as food, shelter, or medical care. |
| Gender | Discrete | Gender. 1: male; 2: female. |
| EL | Discrete | Education levels. 1: junior high school or below; 2: high school or technical secondary school; 3: undergraduate or junior college; 4: master’s or above. |
| MS | Discrete | Marital status. 1: unmarried; 2: married; 3: divorced or widowed. |
| CD | Discrete | Children. 1: no children; 2: one child; 3: more than one child. |
| IC | Discrete | Income. 1: less than 2000 RMB/month; 2: 2000 ~ 5000 RMB/month; 3: 5000 ~ 10000 RMB/month; 4: 10001 ~ 20000 RMB/month; 5: more than 20000 RMB/month. |
| LS | Discrete | Living status. 1: living alone; 2: living with family members; 3: living with others. |
| DK | Discrete | Drinking. 1: no; 2: yes. |
| SK | Discrete | Smoking. 1: no; 2: yes. |
| HMI | Discrete | History of mental illness. 1: no; 2: yes. |
| DA | Discrete | Drug abuse. 1: no; 2: yes. |

**Table S2** The severity levels of depression and anxiety symptoms.

|  | ALL (n = 6105) | Male (n = 3648) | Female (n = 2457) |
| --- | --- | --- | --- |
| PHQ9, depression symptoms | | | |
| Normal | 3824 (62.64) | 2271 (62.25) | 1553 (63.21) |
| Mild | 1545 (25.31) | 903 (24.75) | 642 (26.13) |
| Moderate | 440 (7.21) | 276 (7.57) | 164 (6.67) |
| Moderate severe | 219 (3.59) | 148 (4.06) | 71 (2.89) |
| Severe | 77 (1.26) | 50 (1.37) | 27 (1.10) |
| GAD7, anxiety symptoms | | | |
| Normal | 4260 (69.78) | 2556 (70.07) | 1704 (69.35) |
| Mild | 1361 (22.29) | 779 (21.35) | 582 (23.69) |
| Moderate | 287 (4.70) | 182 (4.99) | 105 (4.27) |
| Moderate severe | 146 (2.39) | 96 (2.63) | 50 (2.04) |
| Severe | 51 (0.84) | 35 (0.96) | 16 (0.65) |

PHQ9, Patient Health Questionnaire-9; GAD7, Generalized Anxiety Disorder-7.

**Table S3** The weighted adjacency matrix for the overall sample in the cross-sectional network.

| Symptoms | EA | PA | SA | EN | PN | CR | ES | OBS | SBS | SU | SES | GAD7 | PHQ9 | ISI | PHQ15 |
| --- | --- | --- | --- | --- | --- | --- | --- | --- | --- | --- | --- | --- | --- | --- | --- |
| EA | 0.00 |  |  |  |  |  |  |  |  |  |  |  |  |  |  |
| PA | **0.45** | 0.00 |  |  |  |  |  |  |  |  |  |  |  |  |  |
| SA | **0.22** | **0.40** | 0.00 |  |  |  |  |  |  |  |  |  |  |  |  |
| EN | **0.07** | 0.00 | 0.00 | 0.00 |  |  |  |  |  |  |  |  |  |  |  |
| PN | **0.14** | **0.12** | **0.05** | **0.43** | 0.00 |  |  |  |  |  |  |  |  |  |  |
| CR | **0.06** | 0.00 | 0.00 | **-0.13** | **-0.08** | 0.00 |  |  |  |  |  |  |  |  |  |
| ES | 0.00 | 0.00 | 0.00 | 0.00 | **0.07** | **0.44** | 0.00 |  |  |  |  |  |  |  |  |
| OBS | 0.00 | 0.00 | 0.00 | **-0.10** | **-0.06** | **0.04** | 0.00 | 0.00 |  |  |  |  |  |  |  |
| SBS | **-0.09** | **0.06** | 0.00 | **-0.14** | 0.00 | 0.00 | 0.00 | **0.34** | 0.00 |  |  |  |  |  |  |
| SU | **0.04** | 0.00 | 0.00 | **-0.09** | 0.00 | **0.16** | **-0.17** | **0.12** | **0.22** | 0.00 |  |  |  |  |  |
| SES | **-0.03** | 0.00 | 0.00 | **-0.10** | **-0.10** | **0.19** | **-0.09** | **0.05** | **0.04** | **0.06** | 0.00 |  |  |  |  |
| GAD7 | 0.00 | 0.00 | 0.00 | 0.00 | 0.00 | 0.00 | 0.00 | 0.00 | 0.00 | 0.00 | 0.00 | 0.00 |  |  |  |
| PHQ9 | **0.04** | 0.00 | 0.00 | 0.00 | 0.00 | 0.00 | **0.03** | 0.00 | **-0.05** | 0.00 | **-0.12** | **0.62** | 0.00 |  |  |
| ISI | 0.00 | 0.00 | 0.00 | 0.00 | **0.04** | 0.00 | **0.08** | 0.00 | 0.00 | 0.00 | 0.00 | **0.04** | **0.28** | 0.00 |  |
| PHQ15 | **0.08** | 0.00 | 0.00 | 0.00 | 0.00 | **0.07** | **-0.05** | 0.00 | **-0.05** | 0.00 | 0.00 | **0.10** | **0.26** | **0.23** | 0.00 |

**Table S4** The weighted adjacency matrix for the males in the cross-sectional network.

| Symptoms | EA | PA | SA | EN | PN | CR | ES | OBS | SBS | SU | SES | GAD7 | PHQ9 | ISI | PHQ15 |
| --- | --- | --- | --- | --- | --- | --- | --- | --- | --- | --- | --- | --- | --- | --- | --- |
| EA | 0.00 |  |  |  |  |  |  |  |  |  |  |  |  |  |  |
| PA | **0.49** | 0.00 |  |  |  |  |  |  |  |  |  |  |  |  |  |
| SA | **0.26** | **0.39** | 0.00 |  |  |  |  |  |  |  |  |  |  |  |  |
| EN | 0.00 | 0.00 | 0.00 | 0.00 |  |  |  |  |  |  |  |  |  |  |  |
| PN | **0.17** | **0.10** | **0.04** | **0.43** | 0.00 |  |  |  |  |  |  |  |  |  |  |
| CR | 0.00 | 0.00 | 0.00 | **-0.16** | **-0.07** | 0.00 |  |  |  |  |  |  |  |  |  |
| ES | 0.00 | 0.00 | 0.00 | 0.00 | 0.00 | **0.57** | 0.00 |  |  |  |  |  |  |  |  |
| OBS | 0.00 | 0.00 | 0.00 | **-0.11** | **-0.05** | 0.00 | 0.00 | 0.00 |  |  |  |  |  |  |  |
| SBS | **-0.08** | 0.00 | 0.00 | **-0.14** | 0.00 | 0.00 | 0.00 | **0.36** | 0.00 |  |  |  |  |  |  |
| SU | 0.00 | 0.00 | 0.00 | **-0.08** | 0.00 | **0.16** | **-0.14** | **0.13** | **0.25** | 0.00 |  |  |  |  |  |
| SES | 0.00 | 0.00 | 0.00 | **-0.10** | **-0.10** | **0.17** | **-0.07** | **0.06** | 0.00 | 0.00 | 0.00 |  |  |  |  |
| GAD7 | 0.00 | 0.00 | 0.00 | 0.00 | 0.00 | 0.00 | 0.00 | 0.00 | 0.00 | 0.00 | 0.00 | 0.00 |  |  |  |
| PHQ9 | **0.03** | 0.00 | 0.00 | 0.00 | 0.00 | 0.00 | 0.00 | 0.00 | **-0.05** | 0.00 | **-0.12** | **0.64** | 0.00 |  |  |
| ISI | 0.00 | 0.00 | 0.00 | 0.00 | **0.06** | 0.00 | **0.06** | 0.00 | 0.00 | 0.00 | 0.00 | **0.05** | **0.26** | 0.00 |  |
| PHQ15 | **0.07** | 0.00 | 0.00 | 0.00 | 0.00 | 0.00 | 0.00 | 0.00 | 0.00 | 0.00 | 0.00 | **0.09** | **0.26** | **0.25** | 0.00 |

**Table S5** The weighted adjacency matrix for the females in the cross-sectional network.

| Symptoms | EA | PA | SA | EN | PN | CR | ES | OBS | SBS | SU | SES | GAD7 | PHQ9 | ISI | PHQ15 |
| --- | --- | --- | --- | --- | --- | --- | --- | --- | --- | --- | --- | --- | --- | --- | --- |
| EA | 0.00 |  |  |  |  |  |  |  |  |  |  |  |  |  |  |
| PA | **0.42** | 0.00 |  |  |  |  |  |  |  |  |  |  |  |  |  |
| SA | **0.15** | **0.36** | 0.00 |  |  |  |  |  |  |  |  |  |  |  |  |
| EN | **0.17** | 0.00 | 0.00 | 0.00 |  |  |  |  |  |  |  |  |  |  |  |
| PN | **0.14** | **0.12** | 0.00 | **0.43** | 0.00 |  |  |  |  |  |  |  |  |  |  |
| CR | **0.07** | 0.00 | 0.00 | **-0.11** | 0.00 | 0.00 |  |  |  |  |  |  |  |  |  |
| ES | 0.00 | 0.00 | 0.00 | 0.00 | 0.00 | **0.28** | 0.00 |  |  |  |  |  |  |  |  |
| OBS | 0.00 | 0.00 | 0.00 | **-0.08** | **-0.09** | **0.10** | 0.00 | 0.00 |  |  |  |  |  |  |  |
| SBS | **-0.11** | **0.09** | 0.00 | **-0.14** | 0.00 | 0.00 | 0.00 | **0.30** | 0.00 |  |  |  |  |  |  |
| SU | 0.00 | 0.00 | 0.00 | **-0.10** | 0.00 | **0.11** | **-0.15** | **0.12** | **0.18** | 0.00 |  |  |  |  |  |
| SES | 0.00 | 0.00 | 0.00 | **-0.09** | **-0.10** | **0.22** | **-0.14** | 0.00 | 0.00 | **0.08** | 0.00 |  |  |  |  |
| GAD7 | 0.00 | 0.00 | 0.00 | 0.00 | 0.00 | 0.00 | 0.00 | 0.00 | 0.00 | 0.00 | 0.00 | 0.00 |  |  |  |
| PHQ9 | **0.05** | 0.00 | 0.00 | 0.00 | 0.00 | 0.00 | 0.00 | 0.00 | **-0.06** | 0.00 | **-0.14** | **0.59** | 0.00 |  |  |
| ISI | 0.00 | 0.00 | 0.00 | 0.00 | 0.00 | 0.00 | 0.00 | 0.00 | 0.00 | 0.00 | 0.00 | 0.00 | **0.27** | 0.00 |  |
| PHQ15 | **0.06** | 0.00 | 0.00 | 0.00 | 0.00 | **0.09** | 0.00 | 0.00 | **-0.07** | 0.00 | 0.00 | **0.11** | **0.28** | **0.23** | 0.00 |

**Table S6** Node centrality measures of strength, closeness, and betweenness in the three cross-sectional networks.

|  | Strength | | |  | Closeness | | |  | Betweenness | | |
| --- | --- | --- | --- | --- | --- | --- | --- | --- | --- | --- | --- |
| Group | ALL | Male | Female |  | ALL | Male | Female |  | ALL | Male | Female |
| PHQ9 | 2.08 | 2.25 | 2.12 |  | -0.25 | -0.42 | -0.37 |  | 1.57 | 1.62 | 1.78 |
| GAD7 | -0.86 | -0.51 | -0.62 |  | -1.22 | -1.07 | -1.18 |  | -1.21 | -1.04 | -0.98 |
| ISI | -1.26 | -0.94 | -1.37 |  | -1.23 | -1.10 | -1.47 |  | -1.21 | -0.92 | -0.98 |
| PHQ15 | -0.49 | -1.03 | -0.03 |  | -0.44 | -1.10 | -0.23 |  | 0.11 | 0.05 | 0.46 |
| EA | 1.25 | 0.99 | 1.26 |  | 0.85 | 0.54 | 0.47 |  | 1.13 | 1.98 | 1.65 |
| PA | 0.36 | 0.43 | 0.53 |  | -0.31 | -0.38 | -0.57 |  | -0.48 | -1.04 | 0.73 |
| SA | -1.25 | -0.89 | -1.35 |  | -1.68 | -1.11 | -1.74 |  | -1.21 | -1.04 | -0.98 |
| EN | 0.52 | 0.68 | 1.06 |  | 1.12 | 1.50 | 1.12 |  | 0.40 | 0.29 | -0.32 |
| PN | 0.67 | 0.65 | 0.08 |  | 0.98 | 1.49 | 0.76 |  | 0.69 | 0.53 | -0.72 |
| CR | 1.07 | 1.16 | 0.49 |  | 1.45 | 1.35 | 1.51 |  | 0.99 | 0.53 | 0.20 |
| ES | -0.09 | -0.15 | -1.10 |  | 0.35 | 0.52 | -0.05 |  | -0.92 | -0.80 | -0.98 |
| SES | -0.74 | -1.22 | -0.32 |  | 0.80 | 0.96 | 1.01 |  | 0.55 | 0.53 | 1.12 |
| OBS | -1.09 | -0.83 | -0.69 |  | -1.17 | -0.99 | -0.18 |  | -1.21 | -1.04 | -0.85 |
| SBS | 0.26 | -0.01 | 0.42 |  | 0.78 | 0.15 | 1.20 |  | 1.13 | 0.77 | 0.60 |
| SU | -0.41 | -0.57 | -0.46 |  | -0.02 | -0.35 | -0.28 |  | -0.33 | -0.43 | -0.72 |

PHQ9, Patient Health Questionnaire 9; GAD7, Generalized Anxiety Disorder 7; ISI, Insomnia Severity Index; PHQ15, Patient Health Questionnaire 15; EA, emotional abuse; PA, physical abuse; SA, sexual abuse; EN, emotional neglect; PN, physical neglect; CR, cognitive reappraisal; ES, expressive suppression; SES, self-esteem; OBS, objective support; SBS, subjective support; SU, support utilization.

**Table S7** The Correlation Stability Coefficient (CS-C) for strength, closeness, and betweenness in three cross-sectional networks.

|  | ALL | Male | Female |
| --- | --- | --- | --- |
| Strength | 0.75 | 0.75 | 0.75 |
| Closeness | 0.67 | 0.59 | 0.44 |
| Betweenness | 0.59 | 0.44 | 0.28 |

**Table S8** The predictability of each node in three cross-sectional networks.

| Symptoms | ALL | Male | Female |
| --- | --- | --- | --- |
| EA | 58.5% | 63.9% | 51.7% |
| PA | 58.7% | 63.6% | 47.2% |
| SA | 46.3% | 51.1% | 33.5% |
| EN | 47.3% | 47.3% | 48.2% |
| PN | 50.7% | 51.0% | 46.9% |
| CR | 35.1% | 46.0% | 22.3% |
| ES | 25.0% | 37.8% | 12.9% |
| OBS | 31.8% | 35.3% | 26.7% |
| SBS | 38.4% | 39.9% | 33.6% |
| SU | 25.9% | 26.1% | 22.5% |
| SES | 29.1% | 29.5% | 29.0% |
| GAD7 | 64.0% | 65.7% | 60.6% |
| PHQ9 | 72.4% | 73.2% | 71.0% |
| ISI | 43.5% | 44.9% | 40.3% |
| PHQ15 | 44.4% | 45.3% | 46.8% |
| Average | 44.7% | 48.0% | 39.5% |

**Table S9** The frequency of each edge in the overall sample Bayesian network.

| From | To | Probability |
| --- | --- | --- |
| Gender | SK | 1.000 |
| Gender | DK | 1.000 |
| Gender | PHQ15 | 1.000 |
| Gender | ES | 1.000 |
| MS | LS | 1.000 |
| SK | DK | 1.000 |
| ISI | PHQ9 | 1.000 |
| PHQ15 | PHQ9 | 1.000 |
| SES | PHQ9 | 1.000 |
| ISI | PHQ15 | 1.000 |
| OBS | EN | 1.000 |
| EA | SA | 1.000 |
| CR | ES | 1.000 |
| EA | PA | 1.000 |
| PA | SA | 1.000 |
| EN | PN | 1.000 |
| EN | SES | 1.000 |
| SU | ES | 1.000 |
| OBS | SBS | 1.000 |
| PN | EA | 1.000 |
| CR | SES | 1.000 |
| OBS | SU | 1.000 |
| SES | ES | 1.000 |
| EN | CR | 1.000 |
| ES | ISI | 1.000 |
| GAD7 | PHQ9 | 1.000 |
| PHQ15 | EA | 1.000 |
| SBS | EN | 1.000 |
| SBS | SU | 1.000 |
| SES | ISI | 1.000 |
| PN | SES | 1.000 |
| Gender | PA | 0.995 |
| EL | IC | 0.995 |
| PHQ15 | GAD7 | 0.995 |
| OBS | PN | 0.995 |
| SU | CR | 0.995 |
| SU | EN | 0.995 |
| SBS | PHQ15 | 0.990 |
| Gender | PN | 0.990 |
| CD | SBS | 0.990 |
| SBS | ISI | 0.985 |
| LS | OBS | 0.980 |
| PN | ISI | 0.965 |
| Gender | SU | 0.955 |
| SES | PHQ15 | 0.920 |
| MIH | DA | 0.905 |

**Table S10** The frequency of each edge in the males Bayesian network.

| From | To | Probability |
| --- | --- | --- |
| MS | LS | 1.000 |
| ISI | PHQ9 | 1.000 |
| PHQ15 | PHQ9 | 1.000 |
| CR | ES | 1.000 |
| SK | DK | 1.000 |
| ISI | PHQ15 | 1.000 |
| PN | EA | 1.000 |
| EN | PN | 1.000 |
| EN | CR | 1.000 |
| EA | PA | 1.000 |
| OBS | SU | 1.000 |
| PA | SA | 1.000 |
| SBS | SU | 1.000 |
| CR | SES | 1.000 |
| GAD7 | PHQ9 | 1.000 |
| OBS | SBS | 1.000 |
| SBS | EN | 1.000 |
| OBS | EN | 1.000 |
| EA | SA | 0.999 |
| SU | ES | 0.999 |
| PN | SES | 0.999 |
| ES | ISI | 0.995 |
| SES | ES | 0.994 |
| CR | SU | 0.991 |
| EN | SES | 0.987 |
| EA | PHQ15 | 0.984 |
| SBS | ISI | 0.984 |
| PHQ15 | GAD7 | 0.980 |
| EL | SK | 0.978 |
| SES | PHQ9 | 0.975 |
| OBS | PN | 0.952 |
| CD | SBS | 0.942 |
| PN | ISI | 0.912 |
| SBS | SES | 0.907 |

**Table S11** The frequency of each edge in the females Bayesian network.

| From | To | Probability |
| --- | --- | --- |
| MS | LS | 1.000 |
| ISI | PHQ9 | 1.000 |
| PHQ15 | PHQ9 | 1.000 |
| EN | PN | 1.000 |
| ISI | PHQ15 | 1.000 |
| EA | PA | 1.000 |
| SES | CR | 1.000 |
| ES | CR | 1.000 |
| PA | SA | 1.000 |
| OBS | SBS | 1.000 |
| PHQ9 | GAD7 | 1.000 |
| SBS | SU | 1.000 |
| EN | SBS | 1.000 |
| SES | PHQ9 | 0.999 |
| SES | PN | 0.999 |
| SU | ES | 0.998 |
| OBS | EN | 0.998 |
| PN | EA | 0.997 |
| SBS | PHQ15 | 0.996 |
| EN | EA | 0.993 |
| EN | SES | 0.992 |
| OBS | SU | 0.988 |
| EA | SA | 0.985 |
| PHQ15 | EA | 0.985 |
| EN | SU | 0.975 |
| OBS | PN | 0.974 |
| LS | OBS | 0.963 |
| CD | SBS | 0.962 |
| SK | DK | 0.954 |
| SU | SES | 0.948 |
| SES | ES | 0.943 |
| CD | KP | 0.943 |
| PHQ15 | GAD7 | 0.933 |
| EN | CR | 0.932 |
| ISI | SES | 0.929 |
| ISI | ES | 0.904 |


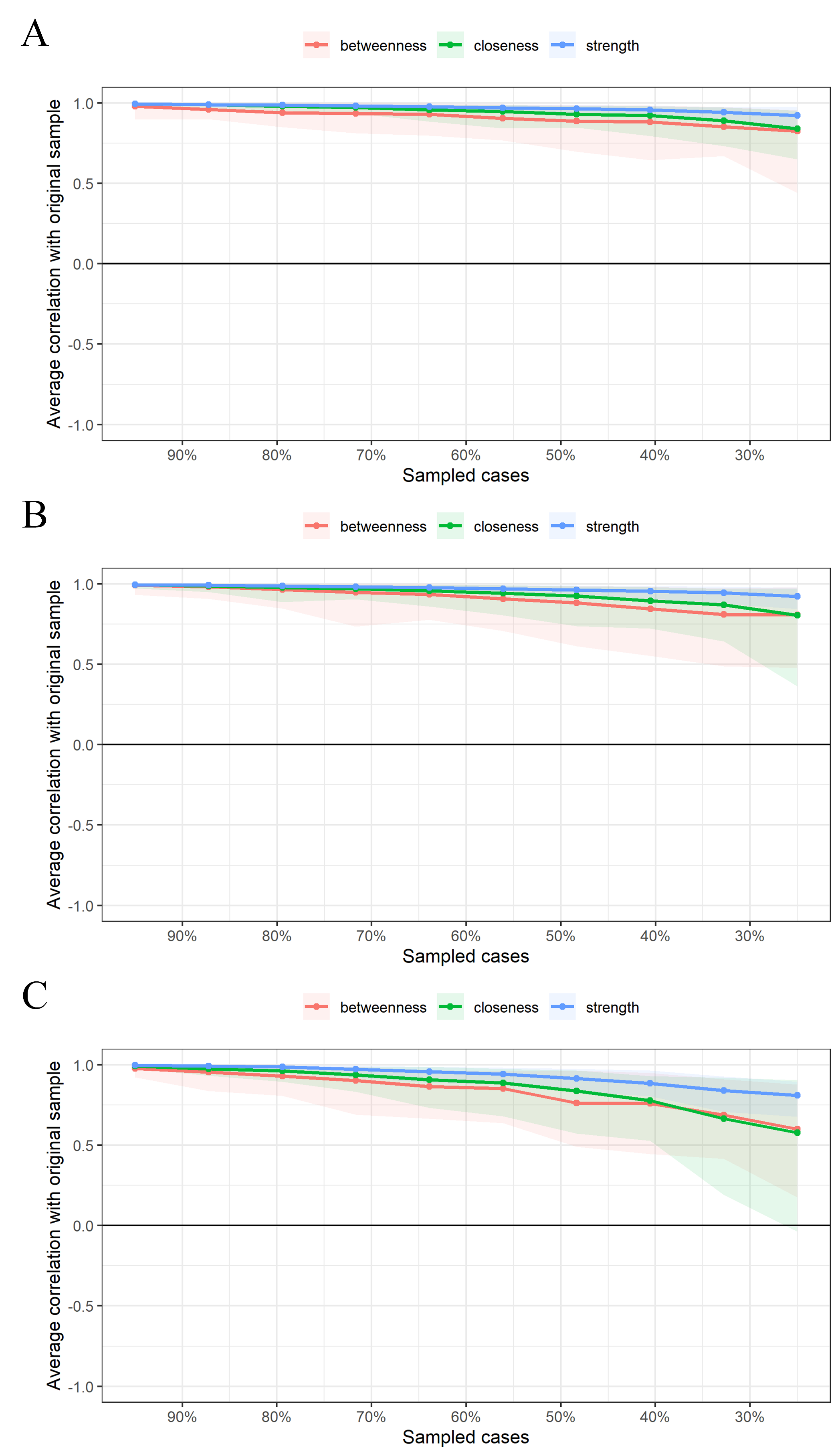


**Fig. S1** Stability of the centrality indices (strength, closeness, and betweenness) in three cross-sectional networks: point estimates with 95% CIs. Lines represent means, and shaded areas indicate the range from the 2.5th to the 97.5th percentiles. (**A**) All participants; (**B**) Males; (**C**) Females.


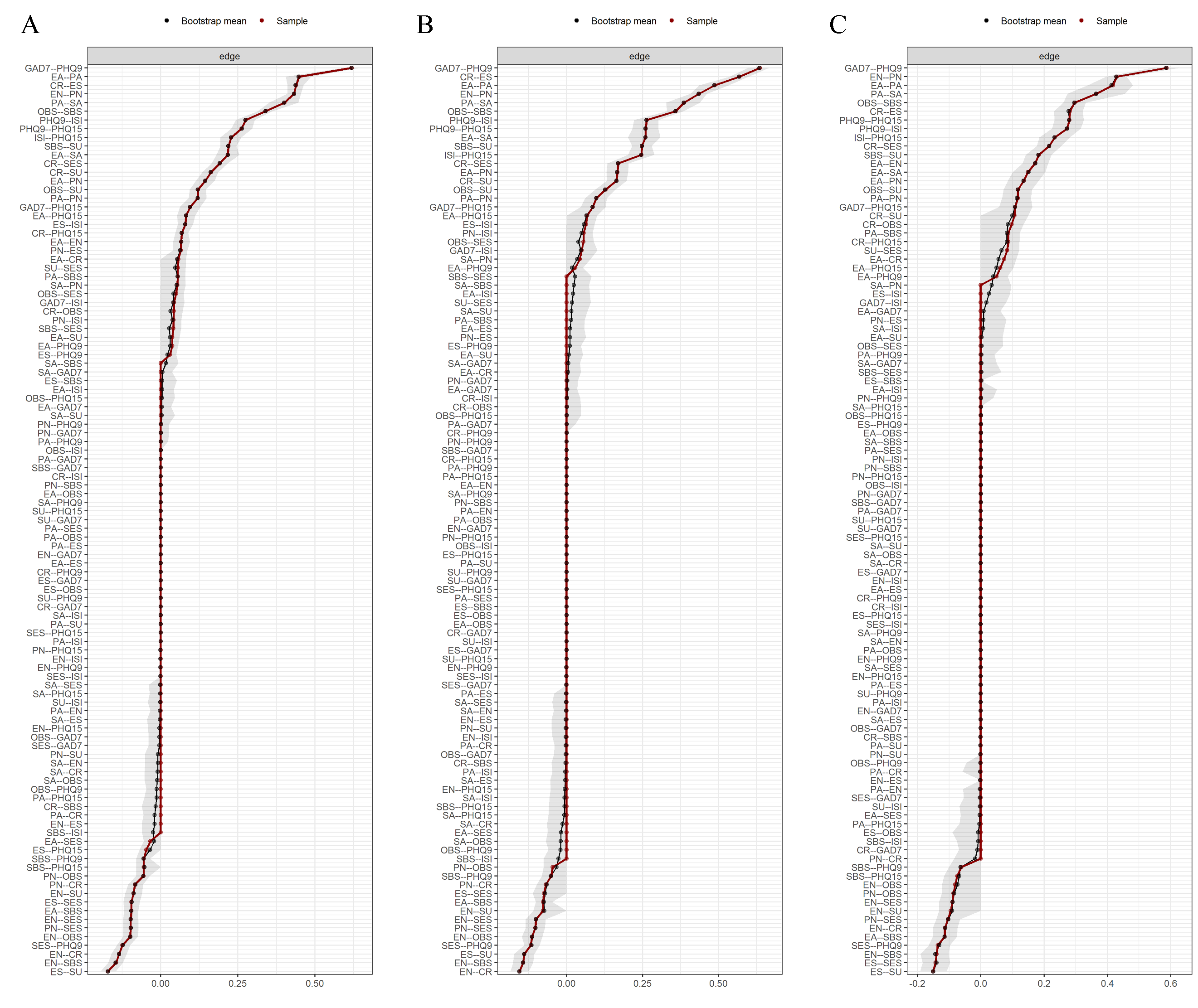


**Fig. S2** The bootstrapped confidence intervals for all edges in three cross-sectional networks. Red dots denote the edge weight values from the original network, while black dots represent the edge weight values obtained through the bootstrap procedure, both arranged in descending order. The gray shaded area corresponds to the 95% confidence intervals of the edge weights, calculated using the non-parametric bootstrap method. (**A**) All participants; (**B**) Males; (**C**) Females.


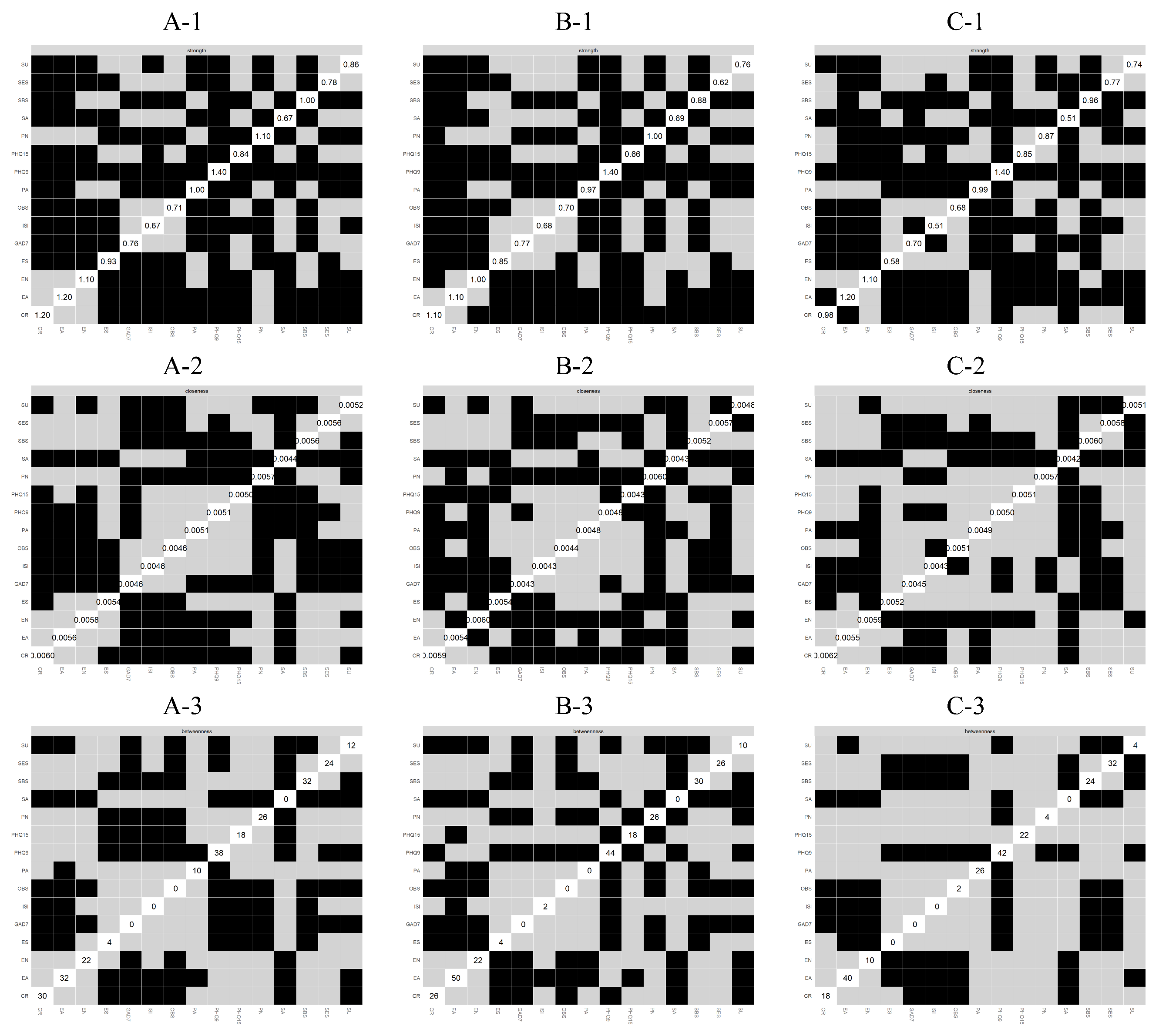


**Fig. S3** The stability test for node strength, closeness, and betweenness in three cross-sectional networks, performed using bootstrapping, displays values within the white boxes along the diagonal line, representing the respective metrics for each node. The box colors indicate the presence or absence of significant differences between symptoms: grey boxes signify no significant differences, while black boxes denote significant differences. (**A-1**) All participants of strength; (**A-2**) All participants of closeness; (**A-3**) All participants of betweenness; (**B-1**) Males of strength; (**B-2**) Males of closeness; (**B-3**) Males of betweenness; (**C-1**) Females of strength; (**C-2**) Females of closeness; (**C-3**) Females of betweenness.


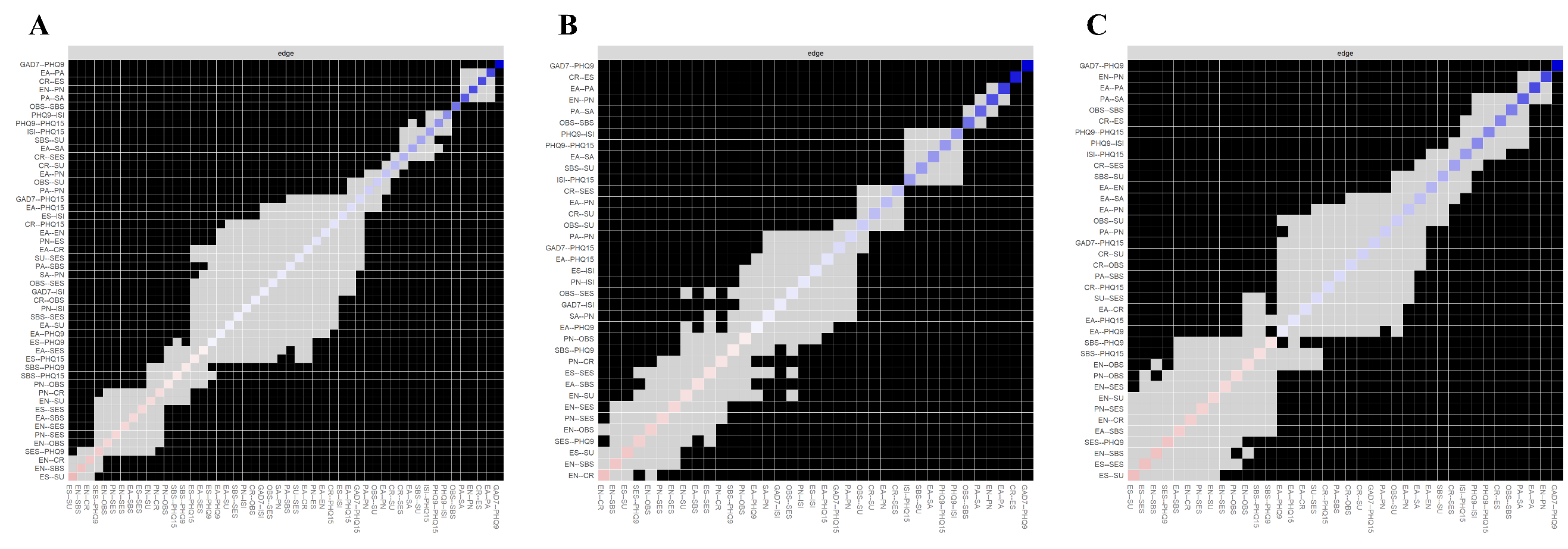


**Fig. S4** The stability test for edge-weight in three cross-sectional networks, conducted using bootstrapping, employs box colors to signify whether edge weights are significantly different (black) or not significantly different (grey). The diagonal line represents the intensity of edge-weights, transitioning from red (indicating negative associations) through white (denoting weaker edges) to blue (representing stronger edge-weights). (**A**) All participants; (**B**) Males; (**C**) Females.
